# Return to Basketball Play Following COVID-19 Lockdown

**DOI:** 10.3390/sports9060081

**Published:** 2021-06-03

**Authors:** Dimitrios I. Bourdas, Emmanouil D. Zacharakis, Antonios K. Travlos, Athanasios Souglis

**Affiliations:** 1 Section of Sport Medicine & Biology of Exercise, School of Physical Education and Sport Science, National and Kapodistrian University of Athens, 41 Ethnikis Antistasis, 17237 Daphne, Greece; 2 Section of Didactics and Coaching in Sport Games, School of Physical Education and Sport Science, National and Kapodistrian University of Athens, 41 Ethnikis Antistasis, 17237 Daphne, Greece; emzach@phed.uoa.gr (E.D.Z.); asouglis@phed.uoa.gr (A.S.); 3 Department of Sports Organization and Management, Faculty of Human Movement and Quality of Life Sciences, University of Peloponnese, Efstathiou and Stamatikis Valioti & Plataion Avenue, 23100 Sparta, Greece; atravlos@uop.gr

**Keywords:** aerobic, fitness training, anaerobic, performance, fatigue, recovery, injury, testing, detraining

## Abstract

Due to concerns regarding the spread of coronavirus (COVID-19), major sporting events and activities have been temporarily suspended or postponed, and a new radical sports protocol has emerged. For most sports there are few recommendations based on scientific evidence for returning to team-game activities following the lifting of COVID-19 restrictions, the extended duration of lockdown, and self-training or detraining in the COVID-19 environment, and this is especially true for basketball. A post-lockdown return to the basketball court ultimately depends on the teams—coaches, trainers, players, and medical staff. Nevertheless, our current scientific knowledge is evidently insufficient as far as safety and return-to-play timing are concerned. This situation presents a major challenge to basketball competition in terms of organization, prioritization, maintaining physical fitness, and decision-making. While preparing an adequate basketball return program, the players’ health is the major priority. In this article we briefly discuss the topic and propose multiple strategies.

## 1. Introduction

The ongoing coronavirus (COVID-19) pandemic has almost brought the world to a halt, having infected more than 157,362,408 people and claimed more than 3,277,834 lives so far (11 May 2021), according to WHO COVID-2019 situation reports [1]. Major sporting events (Olympics, world and national championships, etc.) had to be temporarily suspended following the mandate issued by many countries. During the peak COVID-19 infection phase, physical distancing, self-isolation, and closedown of all sports facilities were imposed. As a result, non-professional and professional athletes, including basketball players, had to lead a restricted lifestyle, being separated from their teams and following individual training protocols. Moreover, the COVID-19 crisis has had an obvious socioeconomic impact on sports, and basketball in particular, at all levels, professional or not [2]. Although home self-quarantine as a way to combat the spread of the infection was imposed by many countries, the impact of this strategy on sports and the consequences of having no group training, especial at non-professional levels, during the COVID-19 crisis have not been sufficiently investigated. It is also understood that since the duration and extent of such restrictions (quarantines) were highly variable worldwide, both across and within regions and countries, obviously that should have different implications on the return to basketball from country to country. Nevertheless, physical fitness and sport skills are expected to deteriorate following imposed restrictions among athletes [3,4,5]. It is a justified speculation that basketball training activities will be no exception and will suffer a significant disruption. We expect lower levels of physical fitness resulting in reduced performance during game-play, increased game-induced fatigue, longer recovery periods following training/games, and increased game-related injury risk in players returning to basketball events after self-training protocols.

Modern basketball requires players to have excellent technical skills and perform medium-to-high-intensity actions that last 15 s as well as explosive muscle actions of high-to-maximal intensity that last up to 2–5 s, in a random order at various recovery intervals, indicating the complexity of the game’s physical demands [6]. Although most of the energy contributing to high-to-maximal-intensity sprint actions derives from phosphocreatine and the fast glycolytic system, aerobic metabolism also plays an important role due to the extended duration of the game [7]. On the other hand, inactivity, detraining, or insufficient training for ~4 weeks degrade all aforementioned technical and physical characteristics and systems [8,9]. Following the COVID-19 lockdown (i.e., a long period of detraining or self-managing training), any abrupt increase in basketball training intensity in order to accelerate biological adaptations and improve physical fitness, in the hope of rapidly reaching pre-COVID-19 levels, would theoretically increase the risk of non-contact injuries [10,11]. In the unprecedented circumstances of a post-lockdown period, basketball reintroduction ultimately depends on basketball players, trainers, coaches, and medical staff. Moreover, there is evidence suggesting current scientific knowledge regarding safety and return-to-play timing is insufficient. In the COVID-19 present (or in any future similar condition or after any significant lay-off), the following general principles can be discerned, based on existing evidence (allowing, of course, for further improvements as more data becomes available).

## 2. Basketball Retraining after a Significant Lay-Off in the COVID-19 Age

### 2.1. COVID-19 Transmission Prophylaxis

Presently, there are no approved treatments for COVID-19 but prevention strategies such as awareness education, physical distancing, personal hygiene, wearing anti-viral facial masks, and vaccination are the best approaches to reduce the risk of COVID-19 infection. For basketball retraining a prime concern should be to eliminate any possibility of COVID-19 transmission from person to person during training sessions. This is not an easy task, but a few simple precautions can be taken [12,13,14,15]. For example, it is strongly advised to sterilize facilities (floor court, benches, etc.) and equipment (balls, weights, etc.) in advance, to ensure that courts have adequate natural ventilation [16] and that special hygiene attention is given to locker rooms and contrast baths. Players’ symptoms and temperature should be checked before each training session. Players must shower with soap and change immediately before and after training or play to reduce contact risk and utilize their own towel always. Players must be educated not to touch their faces, to use hand sanitizers regularly but particularly at substitutions and breaks, and to utilize their own drink bottle at all times. Players must avoid unnecessary physical contact such as hugs, handshakes, high fives, or fan engagement and keep physical distances as much as possible, even on the bench wearing a face mask. A distance of 2 m between individuals is the general medical recommendation, but in basketball it does not seem to be enough [17]. Such a recommendation is based on the assumption that players wear face masks and stand still, and that therefore most of their saliva droplets should reach the floor and/or evaporate before having traveled the distance of 1.5–2 m [17,18]. However, it does not take into consideration the aerodynamic effects introduced by the players’ movement, direction, and acceleration, such as jumping, dribbling, running, or sprinting [18]. Given the above, in order to prevent liquid droplets of asymptomatic (not confirmed and potentially COVID-19-infected) players from being transferred to co-players, exercise drills preferably should not be performed in line, and players should not follow close behind the leading player (i.e., players should not position themselves in the leading player’s potential droplet slipstream). Moreover, in the presence of COVID-19, more specific protective measures should be added to those proposed by the World Health Organization, Centers for Disease Control and Prevention and any national public health guidelines [19]. Here are some advisable precautions: (1) from the start of the post-lockdown training sessions, teams should be split into smaller groups (*n* ≤ 8); (2) although the use of face masks by healthy individuals remains a controversial subject and there is little, if any, evidence of their effectiveness when used by players [20], they could be used as a preventive measure during team training, if they can be tolerated, until more evidence becomes available; (3) exercise drills should be performed in a side-by-side arrangement; and (4) whenever there is a need for increased running speed, players should keep larger distances between them. Since basketball is a high-risk contact sport, players need to learn to identify the COVID-19 symptoms so that timely testing can be conducted, or if permissible, players should be tested weekly or more often as part of regular routines. It is also worthy to notice that antigen test results should always be interpreted in the context of the player being tested’s exposure history and clinical presentation, and a polymerase chain reaction test (PCR, which is considered the “gold standard” in COVID-19 detection) should be used to confirm a positive antigen result in an asymptomatic individual with no known exposure.

### 2.2. Principles of Basketball Training after a Significant Lay-Off

After a significant lay-off and before resuming training, coaches will have to consider the following: (1) the duration of the lockdown or detraining period in conjunction with a player’s physical condition is an important factor in tailoring the retraining strategy to individual needs; (2) appropriate fitness tests and measurements (Table 1) are needed for a valid evaluation of the players’ physical fitness [21,22,23]; and (3) how much time they have until the onset of the next game, especially for professional players. Accordingly, due to time constraints, aerobic and anaerobic training should be introduced gradually, building to basketball-specific high intensity drills and should include different types of exercise and drills in combination with applied technical and tactical skills. Assuming that lockdown with a limited possibility to train for >4 weeks (~20–40% workload of a normal competitive training period) has caused a greater decline in basketball-specific fitness than a normal basketball off-season period of ~2–3 weeks [24], the recommended return time to normal intensity and volume training, minimizing the risk ratio of non-contact injury, is estimated at 3–5 weeks [11].

Generally, the reported distance covered by basketball players ranges from 4.5 to 5 km [25,26], and an aerobic capacity of 42–60 mL·kg^−1^·min^−1^ for male basketball players (or up to 65 mL·kg^−1^·min^−1^ for elite players) appears quite sufficient to meet these demands [27,28,29]. The corresponding VO_2max_ and HR_max_ values for a basketball game are ~65% and 84%, respectively, and for a training session they are 69% and 84%, respectively [26,30]. Consequently, the aerobic training program should have an intensity close to ~70–100% of age-predicted HR_max_ (Table 2) [31]. Therefore, the return to basketball training should be gradual, integrating moderate-to-high-intensity aerobic exercises combined with basic strength training, adapted to specific basketball demands and also depending on the individuals’ bio-physiological profile and basketball position [32,33,34].

**Figure 1:**
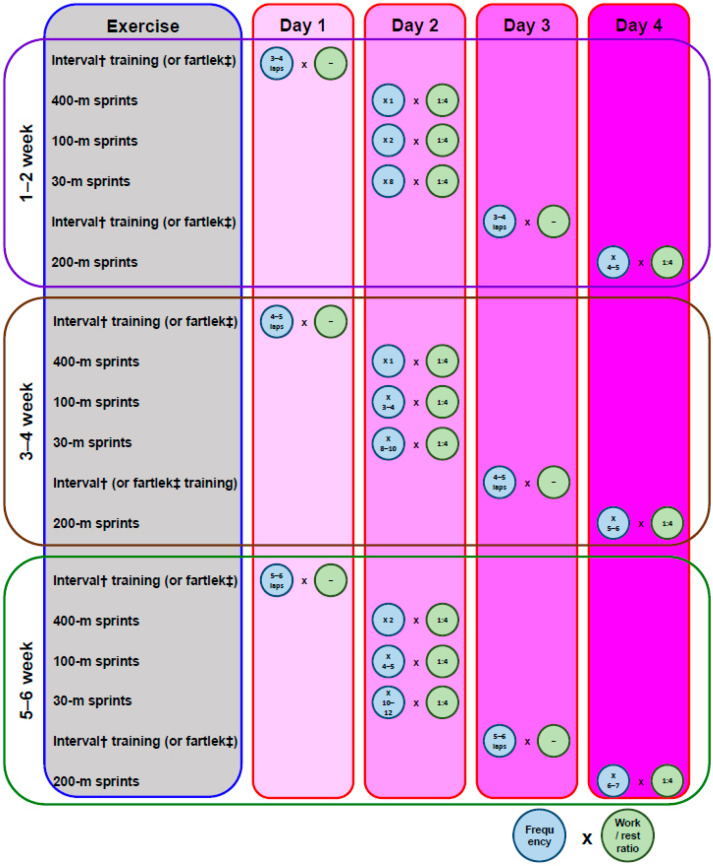
Example of a periodized 6-week preseason anaerobic conditioning program (modified by Hoffman and Maresh [28]). ^†^ Sprint 100 m and jog the turns (continuous lap performance); ^‡^ interspersed high-intensity sprints with lower-intensity running.

Basketball performance relies on the player’s anaerobic capacity to sustain high-intensity movements, sprints, and offensive and defensive transitions. A typical anaerobic conditioning program could be performed 3–4 days/week, with gradually increasing intensity and volume (Figure 1). Although a 5 vs. 5 player ratio in training games is preferable in order to achieve game-specific dynamics, under the circumstances this is just not possible due to the increased risk of cross-infection (from a potential asymptomatic and not confirmed COVID-19 infected player). However, the best way to build up basketball physical fitness is to use integrated training drills on the basketball court. In that context, small side games (e.g., 4 × 4 min, 2 vs. 2 with 3 min passive recovery), ball drills, and repeated-sprint-ability training (e.g., 3 × 6 × 20 m shuttle running with 20 s and 4 min recovery) should have a positive impact on technical skills while simultaneously improving the players’ physical performance [35,36,37]. It is also evident that increasing the relative playing area or keeping it the same, while reducing the number of basketball players involved or keeping their number stable, could cause a remarkable increase in the physiological load [37,38,39,40]. For instance, during a 2 vs. 2 small side game, 92.06 ± 5.6% of mean HR_max_ has been documented [41].

**Figure 2:**
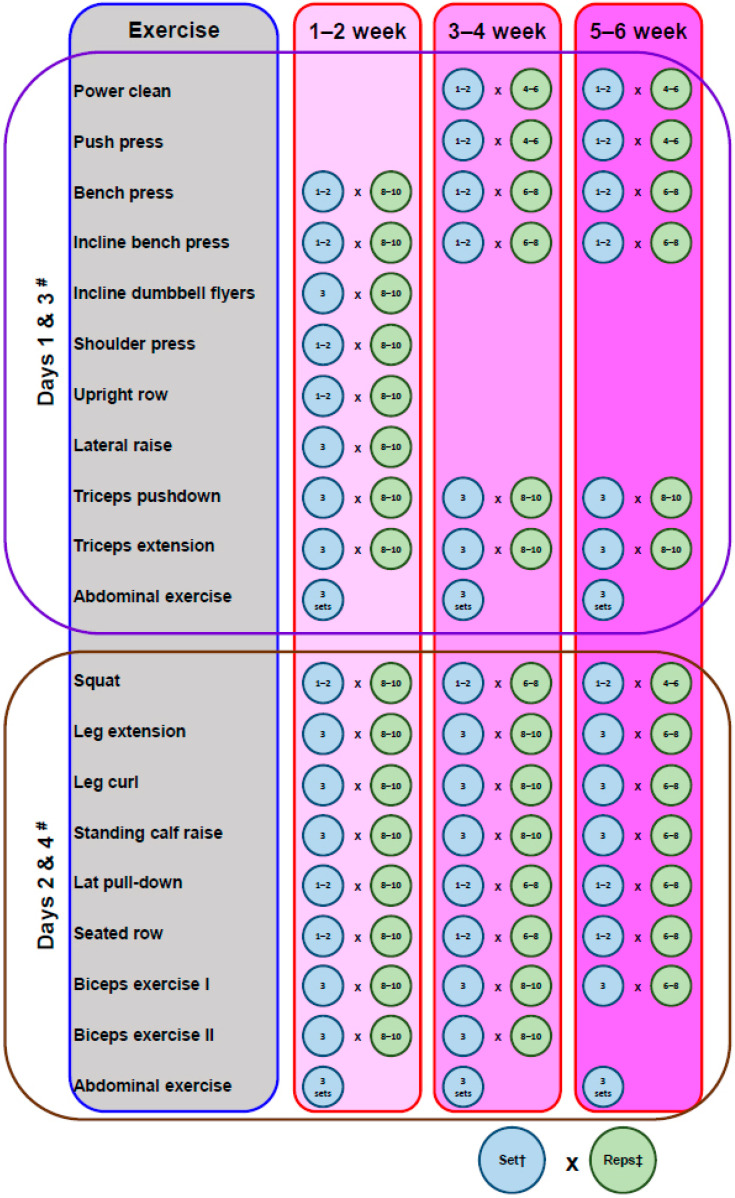
Example of a periodized preseason basic power training program for basketball players (modified by Hoffman and Maresh [28]). ^#^ Two times per week, not consecutive days; ^†^ set number subjected to the player’s level; ^‡^ repetition maximum.

Pre-COVID-19-achieved strength gains could be easily maintained during <4 weeks of detraining [42]. However, sprints, jumping, direction changes, sideways displacements, and many other fundamental basketball movements depend on power-related features; therefore, strength training should be recommended (Figure 2) but not to complete muscle exhaustion, so as to avoid the risk of possible immunosuppressive side effects [43]. It should be noted that a reduced-strength training program or detraining contribute very little to retaining explosive strength when practicing regular basketball [44]. Moreover, plyometrics could also be gradually introduced into the training process with caution, if the players’ vertical jump or overall power and change of direction speed ability needs immediate improvement [44].

**Figure 3:**
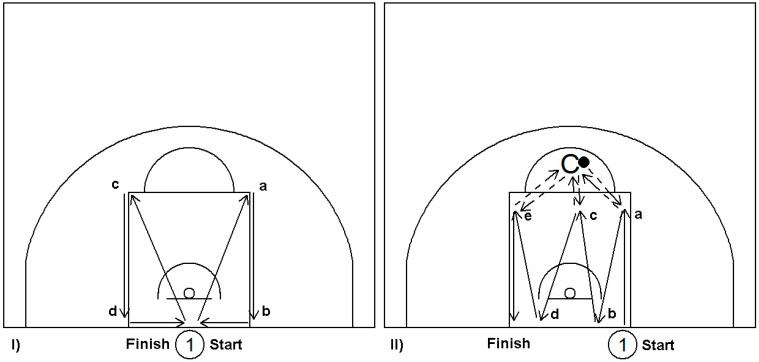
Two examples of agility and running-technique drills (modified by Anderson [48]). The player should always focus on minimizing the time to finish the drill and change direction. Drills are repeated 4–8 times, with a 60–90 s rest in between, depending on the player’s level. (**I**) Left with no ball. Player starts beneath the basket and moves from (a), (b), (c), and (d) points to the finish/start point. Player movements could be various combinations of backpedals with hands in a raised defensive position, backwards run, lateral steps keeping hands raised in the defensive position, leap to touch the net or backboard or the hoop ring, etc. (**II**) Right with ball. Similarly, player starts on the baseline facing the court and moves from (a), (b), (c), (d), and (e) points to the finish point. Player movements could be various combinations of dribbling, sprinting, jumps (to touch the net, or the backboard, or the basket ring), passing (chest-passes, overhead-passes), pass receiving, etc. Arrows indicate player movement, dashed arrows indicate passes, the numbered cycle indicates player position, the black dot indicates the ball, and C indicates coach position.

Agility is a prerequisite for successful basketball performance and is affected by speed, strength, coordination, balance, and flexibility [45,46]. Again, it is important to practice agility drills on the basketball court as part of the integrated training with basketball applied drills (Figure 3) and small-side games [47,48].

Basketball also requires a high degree of flexibility. Sufficient stretching sessions reduce muscle-related injuries, increase the joints’ range of motion, and minimize the possibility of subsequent athletic performance impairments [49]. Therefore, dynamic stretching exercises before games are recommended, and basketball-specific actions that involve the major muscle groups should be included in the daily training routine. Static stretching performed separately will improve joints’ ranges of motion and flexibility. Moreover, structured warm-up and basketball-circuit-related drills will further facilitate the player’s gradual return to training sessions.

During the COVID-19 period, restriction measures and physical distancing undoubtedly have made quality basketball training quite difficult to achieve [50,51]. This drawback will potentially affect not only the players’ game performance in the near future, but also their post-game recovery, especially if too many games are scheduled within a short period in order to finish the season in time. Under these unprecedented conditions, the players’ biological workload will be burdened in proportion to the time available for compensating for the lost aerobic capacity. With regard to professional players, delayed onset of post-training muscle soreness (DOMS) and post-game residual fatigue should be regularly monitored by resting heart rate, RPE, and biochemical markers of muscle damage (e.g., total antioxidant capacity, creatine kinase, reduced glutathione, oxidized glutathione, myoglobin concentration, protein carbonyls, lactate dehydrogenase) in order to prevent physical impairment, ensure optimum recovery, and minimize the possibility of training disruption [52,53,54,55,56,57,58]. Post-training or post-game stretching combined with compression garments, adequate rest, and sleep are also highly recommended for optimum recovery [54,59,60,61,62,63,64,65].

Apart from any anti-COVID-19 strategy, nutrition and hydration are essential to life and health. Thus, proper hydration and a balanced diet rich in micronutrients and carbohydrates are generally recommended. Moreover, several nutraceuticals (e.g., probiotics, vitamin D, selenium, vitamin C, zinc, cinnamaldehyde, curcumin, quercetin, lactoferrin) have immunomodulatory function, antioxidant activity, and proven antimicrobial, antiviral, and anti-inflammatory effects [66,67]. These phytonutrients may have a feasible role in preventing COVID-19 infection and supporting infected players, but scientific evidence based on the associations of micronutrients and COVID-19 are lacking [68,69]. On balance, a combination of some of the aforementioned vitamins and minerals in the form of a food supplement may assist players to boost their immune system and prevent the infection from progressing to a severe stage, providing preventative and therapeutic assistance against COVID-19 [67,69,70].

Furthermore, the challenges of players during the COVID-19 are not limited only to their physical fitness and technical skills recovery. Team players have to deal with many psychological problems, such as lack of communication with teammates, feelings of isolation [71], high levels of perceived stress, and maladaptive psychobiosocial states [72]. Given that lockdown has wide-ranging, substantial, and potentially acute or/and long-term psychological effects (e.g., depression, anxiety, adverse behaviors, smoking, alcohol use, eating and sleep disorders) [73,74], identifying and controlling these consequences should be also a priority for players and coaches, which should motivate the players to seek professional assistance and social support when it is needed [75,76,77]. As mental health and mental preparation of basketball players should not been ignored [78], players may benefit from psychological interventions provided by sport psychologists on cognitive–emotional regulation strategies [79], and develop psychological skills like stress management, attentional focus, communication, goal setting, mental practice, self-talk, and confidence in order to contribute to team performance efficiency and effectiveness [80,81].

COVID-19 vaccination campaigns are under way across the world, COVID-19 is showing signs of recession worldwide, and restrictions drop each passing day. However, positive cases are still increasing in many countries, therapeutic agents are not available for COVID-19, and experts are concerned about the vaccine’s efficacy and safety against new mutations. Under these unparalleled scenarios, the future of team sports, such as basketball, is at stake. Therefore, the above recommendations for a return to basketball are not only practical in the current context but can also be applied after any significant lay-off in the future or even during the off-season, where players are less physically active anyway.

## 3. Conclusions

As new COVID-19 cases continue to emerge throughout the world, many healthy players are asked to stay at home in self-quarantine. In some countries, fitness centers and sport facilities where players normally train remain temporarily closed. The principles described in this article could help players to reach peak performance after a significant lay-off. They will also enable coaches and trainers (Table 3) to deliver a better on-field training load, either external (e.g., distance performance at variant intensities [82]) or internal (e.g., RPE, oxygen consumption, heart rate [34]). Integrated basketball training and small-side games seem ideal for the time available and meet the multifactorial physiological demands of basketball. Exhaustive training or abrupt increase of non-basketball-specific training and/or workload may either lead to overtraining, with an increased risk of non-contact injuries and reduced function of the immune system [10,11,43], or make the smooth transition to basketball-specific movements harder.

## Figures and Tables

**Table 1:** Example, with proposed order, of test battery for basketball (modified from McGuigan [21] and Wen et al. [22]).

Test Order	Test Description	Tests	Physiological Variable Tested
1	Anthropometric measures	BMI or SKF measurements, flexibility	Body composition, flexibility
2	Change of single (or multiple) direction(s)	T-test, lane agility	Speed, agility
3	Muscle strength, maximum muscular power, and non-exhausting power tests	One-repetition maximum squat and bench press, isokinetic dynamometry, CMJ, one-step jump	Maximum strength, power
4	Explosiveness measures	5 m, 10 m, and ¾ court sprints	Speed/acceleration
5	Muscle fitness	Push-up, sit-up	Muscular endurance
6	All out anaerobic fitness	Wingate, suicide runs, shuttle runs	Anaerobic power
7	Cardiovascular/endurance	VO_2max_, Yo-Yo IR2, Yo-Yo IE2	Aerobic capacity

Abbreviations: BMI, body mass index; CMJ, counter movement jump; SKF, skinfold; VO_2max_, maximal oxygen uptake; Yo-Yo IR2, Yo-Yo intermittent recovery level 2; Yo-Yo IE2, Yo-Yo intermittent endurance level 2.

**Table 2:** Principles of aerobic training in basketball (modified by Bangsbo et al. [31]).

Aerobic Training	HR *, (Mean bpm (Range))	HR_max_ *, (Mean % (Range))	Running Speed, (Mean km/h (Range))	RPE ^†^, (Mean (Range))
Low intensity	130 (100–160)	65 (50–80)	11 (9–13)	2 (1–3)
Moderate intensity	160 (140–180)	80 (70–90)	14 (12–16)	4 (3–5)
High intensity	180 (170–200)	90 (85–100)	17 (15–19)	6 (5–7)

* If HR_max_ = 200 bpm; ^†^ scale 1–10; abbreviations: HR, heart rate; HR_max_, maximum heart rate; RPE, rate of perceived exertion scale.

**Table 3:** Coaches’ and trainers’ recommendations overview for basketball return after a significant lay-off in the COVID-19 era.

	General Recommendations for Basketball Return
i	Apply appropriate anti-COVID-19 precautions according to national public health guidelines.
ii	Evaluate players’ physical conditions.
iii	Design training microcycles/schedules beforehand and take into account the lockdown or detraining duration and the time until the onset of the next gaming schedule in conjunction with players’ physical condition.
iv	Integrate moderate to high intensity aerobic exercise in combination with basic strength training, tailored to specific basketball requirements and also depending on the bio-physiological profile and position of each individual.
v	Integrate basketball training drills and small-side games to meet the multifactorial physiological demands of the sport.
vi	Integrate agility drills on the basketball court.
vii	Gradually increase the training volume and intensity.
viii	Avoid unnecessary exhaustive strength training or abrupt increase of non-basketball-specific training and/or workload.
ix	Introduce sufficient stretching sessions post-training or post-game combined with compression garments.
x	Adopt mental strategies for mental preparation, building resilience and mental health.
xi	Promote adequate rest, sleep, proper hydration, and a balanced diet rich in micronutrients, carbohydrates, and vitamin D.
xii	Monitor as possible players’ RPE, DOMS, and biochemical markers of muscle damage and accordingly modify your training if there is a need.

Abbreviations: DOMS, delayed onset muscle soreness; RPE, rate of perceived exertion.

## Data Availability

No new data were created or analyzed in this study. Data sharing is not applicable to this article.
